# Performance assessment of aquatic macrophytes for treatment of municipal wastewater

**DOI:** 10.1186/2052-336X-12-106

**Published:** 2014-07-16

**Authors:** Mumtaz Shah, Hashim Nisar Hashmi, Arshad Ali, Abdul Razzaq Ghumman

**Affiliations:** 1 Department of Civil and Environmental Engineering, UET, Taxila, Pakistan; 2 Department of Civil Engineering, UET, Taxila, Pakistan; 3 Civil Engineering Department, MCE, Rasalpur, Pakistan

**Keywords:** Aquatic macrophytes, BOD_5_, Duckweed, Phytoplankton, NEQS, Plant growth, Waste stabilization ponds

## Abstract

The objective of the study was to evaluate the performance of three different aquatic macrophytes for treatment of municipal wastewater collected from Taxila (Pakistan). A physical model of treatment plant was constructed and was operated for six experimental runs with each species of macrophyte. Every experimental run consist of thirty days period. Regular monitoring of influent and effluent concentrations were made during each experimental run. For the treatment locally available macrophyte species i.e. water hyacinth, duckweed & water lettuce were selected to use. To evaluate the treatment performance of each macrophyte, BOD_5_, COD, and Nutrients (Nitrogen and Phosphorus) were monitored in effluent from model at different detention time of every experimental run after ensuring steady state conditions. The average reduction of effluent value of each parameter using water hyacinth were 50.61% for BOD_5_, 46.38% for COD, 40.34% for Nitrogen and 18.76% for Phosphorus. For duckweed the average removal efficiency for selected parameters were 33.43% for BOD_5_, 26.37% for COD, 17.59% for Nitrogen and 15.25% for Phosphorus and for Water Lettuce the average removal efficiency were 33.43% for BOD_5_, 26.37% for COD, 17.59% for Nitrogen and 15.25% for Phosphorus. The mechanisms of pollutant removal in this system include both aerobic and anaerobic microbiological conversions, sorption, sedimentation, volatilization and chemical transformations. The rapid growth of the biomass was measured within first ten days detention time. It was also observed that performance of macrophytes is influenced by variation of pH and Temperature. A pH of 6-9 and Temperature of 15-38°C is most favorable for treatment of wastewater by macrophytes. The option of macrophytes for treatment of Municipal sewage under local environmental conditions can be explored by further verifying the removal efficiency under variation of different environmental conditions. Also this is need of time that macrophyte system should be used for treatment of wastewater because their performance is comparable to conventional wastewater treatment plants and also the system has very low O&M costs.

## Introduction

Wastewater is any liquid that has been adversely affected in quality by anthropogenic influence. It comprises liquid waste discharged by domestic residences, commercial properties, industry, or agriculture and can encompass a wide range of potential contaminants and concentrations. In the most common usage, it refers to the municipal wastewater that contains a broad spectrum of contaminants resulting from the mixing of wastewater from different sources. Urban wastewater contains 99% water, and other materials make up the portion. The potential pollutants include pathogens, oil and grease, metals, organic matter (OM), solids and nutrients such as Nitrogen (N) and Phosphorous (P). The actual proportion of each constituent within any given wastewater varies depending on the spatial and temporal differences [1].

In recent years, the amount of wastewater produced from several activities has increased as a result of the rapid improvement of living standards [2]. Although some communities treat their wastewater in a suitable way, others lack convenient treatment systems, thus discharging untreated wastewater into the natural environment. Pollutants (e.g. heavy metals) enter aquatic systems via numerous pathways, including effluent discharge, urban and agricultural run-off. Contaminants present in sewage commonly include a wide range of metallic and organic compounds [3].

Wastewater treatment technology needs to be appropriate and sustainable. It also needs to be less costly, easy to operate and maintain, and very efficient in removing both organic matter and heavy metals. In developing countries natural treatment systems, are more suitable. Natural treatment systems are considered one of the best treatment options, particularly in warm climates [4]. Wetlands with macrophytes are one of the many types of natural systems that can be used for treatment of municipal wastewater. According to Trepanier, a wetland specifically constructed for the purpose of pollution control and waste management, at a location other than existing natural wetlands” is known as constructed wetland. Wetlands have many unique benefits as a wastewater treatment process, including the ability to operate on ambient solar energy, self-organize and increase treatment capacity over time, rich in biodiversity, produce oxygen and consume carbon dioxide, and achieve high levels of treatment with minimum maintenance [5]. Macrophytes have been used effectively to treat different types of wastewaters. This is mainly due to their nutrient absorbing capacity, simplicity, low construction/operation and maintenance cost, low energy demand, process stability, potential benefits of the harvested materials [6].

The macrophytes have several properties in relation to the treatment processes. The most important effects of the macrophytes in relation to the wastewater treatment processes are the physical effects of the plant tissues give rise to filtration effect and provide of surface area for attached microorganisms. The pollutants removal of macrophytes by plant uptake and oxygen release affects the wastewater treatment processes in different extends. The macrophytes provide habitat for wildlife [7].

Zhang et al. [8] conducted investigation regarding the efficiency of macrophytes based treatment system in China for Municipal Wastewater Treatment. According to his findings Large-scale centralized wastewater treatment systems often prevail in industrial countries and have been regarded as a successful approach during the last century [8].

According to Mayo et al. [9] the removal of faecal coliforms was investigated in pilot-scale water hyacinths ponds. The investigation was conducted to evaluate the role of solar intensity, pH, dissolved oxygen, temperature, sedimentation, and attachment of faecal coliforms on Eichhornia crassipes on disappearance of bacteria in water hyacinths ponds. The results showed that environmental factors such as solar intensity and pH were the key factors when water hyacinths ponds have a large exposed surface area [9].

Water pollution is becoming a serious issue of the entire world due to the rapid population growth, unsuitable treatment technology and inadequate management. In Pakistan untreated municipal wastewater is indiscriminately discharged into water bodies. Rapid urbanization and industrialization have resulted in increased pollution load in the rivers and streams. In large cities municipal wastewaters from almost whole city along with commercial/industrial effluents is being discharged into water bodies in the immediate vicinity of the city (i.e. rivers, surface drains & canals). As a result pollution level in the water bodies is ever increasing due to the increase in the population and commercial/industrial development. There are several sophisticated treatment systems available, such as activated sludge process, rotating biological contactor, and aerated lagoon, but they require high capital, operational, and maintenance costs. In Pakistan treatment plant based of trickling filter was installed in Karachi while other based on activated sludge process was installed in Islamabad but both the treatment plant are non functional now a days due to high maintenance and no skilled staff availability. Waste stabilization ponds are currently functioning in Peshawar. due to the experience of wastewater treatment plants, biological treatment system with low operational and capital costs is preferred especially for developing countries like Pakistan, with warm climate all year round. Pakistan has sufficient land for natural wastewater treatment technology in the outskirts of cities. Thus, the maximum advantages of climate and land availability should be taken for wastewater treatment purpose. The attractive method is combination of waste stabilization pond along with macrophytes. The mechanism of treatment in this system is same as in constructed wetland. This method has been used as the effective low-cost technologies that require minimum energy to operate that are suitable for urban as well as for rural areas in Pakistan.

Though, the treatment of wastewater by macrophyte plants has been started long before. The question how low aquatic plants can decrease the wastewater quality indicators still remain unanswered. Mainly macrophytes treat wastewater by organic matter uptake from the wastewater. at the roots of macrophytes small zones exist which arrest organic matter from wastewater. It is of interest to determine the lower bounds of pollutant contents that can reached because of their removal by aquatic plants and under what condition removal occur. This reflects on the range of application of aquatic plants (macrophytes) for wastewater treatment. Therefore, a physical Macrophyte based treatment plant model was constructed to treat municipal wastewater from University of Engineering& Technology (UET), Taxila. Wastewater was discharged into physical model containing Macrophytes. The objectives of the study aimed to evaluate the removal performance of pollutants as COD, BOD_5_ and nutrients (N&P) by different macrophytes species.

## Material and methods

### Treatment system

**Figure 1:**
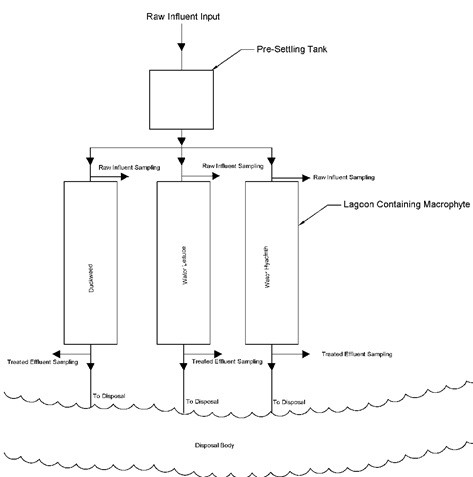
Experimental setup for the study.

A circular storage tank (having volume of 2280 L) was used for collection of municipal wastewater from university sewer. Form that storage tank raw wastewater was distributed to individual model compartments (1.52 m (L)×1.83 m (W)×0.91 m (D)) containing different macrophyte species. Figure 1 shows the layout of the experimental setup.

### Municipal wastewater

The municipal wastewater for the study was collected from the municipal sewer of UET Taxila containing wastewater of university and university colony.

### Selection of species of macrophytes

The selection of macrophytes for the study was done on the availability of macrophytes locally as well as the environmental conditions of the area. Keeping in view of all the factors three macrophytes i.e. Water Hyacinth, Duckweed & Water Lettuce were selected.

### Analytical procedure

The study was carried for 18 months in physical model. The BOD_5_, COD, and ammonia, Phosphorus were analyzed according to Standard Methods for the Examination of Water and Wastewater.

## Results and discussions

The removal of pollutants by macrophytes may occur through a number of processes, including sedimentation, filtration, plant uptake/removal efficiency, adsorption, formation of solid compounds, and microbial-mediated reaction.

### Raw wastewater characteristics

The wastewater for study has been collected from main foul sewer line of UET Taxila, Pakistan. The characteristics of the untreated wastewater is described Table 1;

**Table 1:** Characteristics of raw wastwater

**Sr. #**	**Parameter**	**Unit**	**Value**
1.	BOD_5_	mg/l	132
2.	COD		236
3.	Nitrogen		2.65
4.	Phosphorus		2.1

### BOD_5_

**Figure 2:**
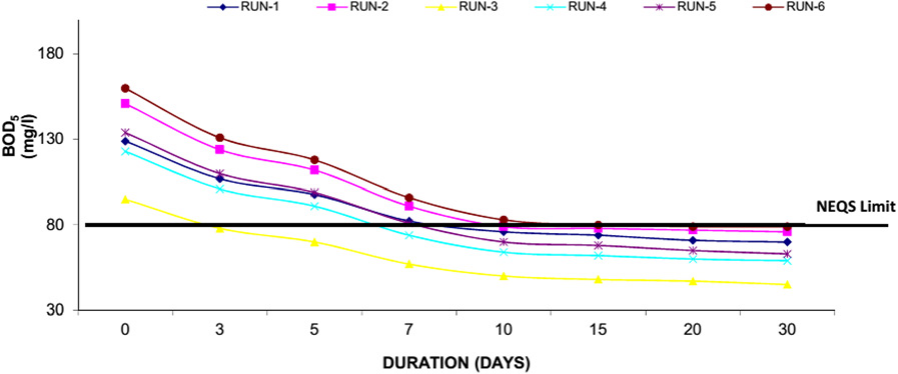
**Reduction of BOD**_**5 **_**with water hyacinth.**

**Figure 3:**
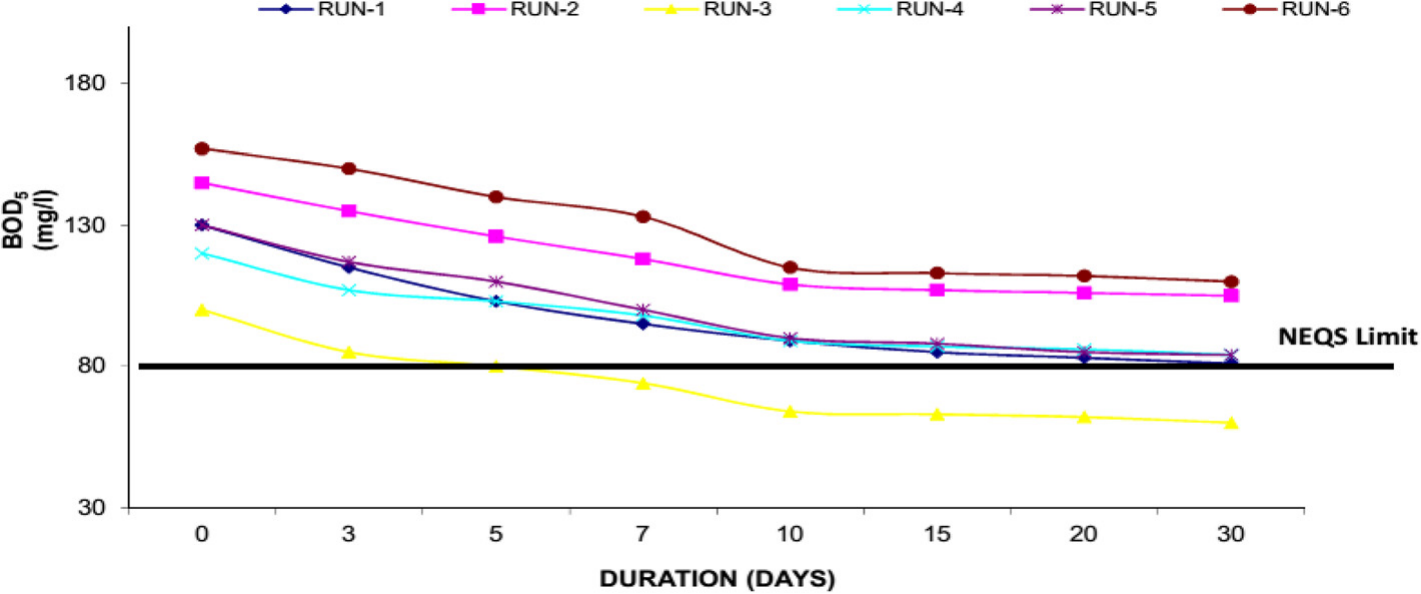
**Reduction of BOD**_**5 **_**with duckweed.**

**Figure 4:**
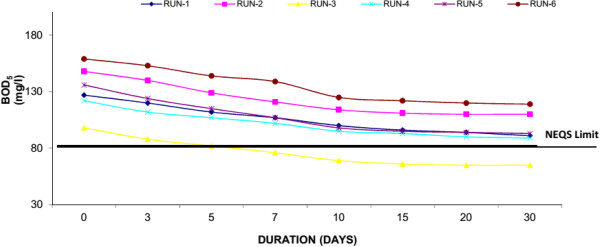
**Reduction of BOD**_**5 **_**with water lettuce.**

Biochemical oxygen demand (BOD_5_) is a measure of the oxygen consumption of microorganisms in the oxidation of organic matter. Settleable organics are rapidly removed in experimental system by quiescent conditions, deposition, and filtration. Attached and suspended microbial growth is responsible for removal of soluble BOD_5_. The influent concentrations were ranged in 95-160 mg/l showing the medium strength sewage. The BOD_5_ effluent concentrations at 30th day of each experiment run for Water Hyacinth system were 70 mg/l (Run-1), 76 mg/l (Run-2), 45 mg/l (Run-3), 59 mg/l (Run-4), 63 (Run-5) and 79 mg/l (Run-6). Where for Duckweed based system showed 81 mg/l (Run-1), 105 mg/l (Run-2), 60 mg/l (Run-3), 84 mg/l (Run-4), 84 (Run-5) and 110 mg/l (Run-6) at 30th day of each experimental run. Similarly Water Lettuce showed, 91 mg/l (Run-1), 110 mg/l (Run-2), 65 mg/l (Run-3), 89 mg/l (Run-4), 93 (Run-5) and 119 mg/l (Run-6) at 30th day of each experimental run. The results showed that water hyacinth showed the maximum removal (i.e. 50.61% average reduction) of BOD_5_ as compared to Duckweed and Water Lettuce. Figures 2, 3 & 4 shows removal of BOD by water hyacinth, Duckweed and Water Lettuce respectively. It was observed that removal of BOD is mainly in first 10 days of each Experimental Run, after that the removal is at a slower rate. It is mainly due to plant update is much high in first 10 days similarly significant plant growth was observed during this period as well. In the study it was also found that the desirable concentration of BOD_5_ (i.e. <80 mg/l) as prescribed by National Environmental Quality Standards (NEQS, Pakistan) was achieved in water hyacinth system at 10th day in each run.

**Figure 5:**
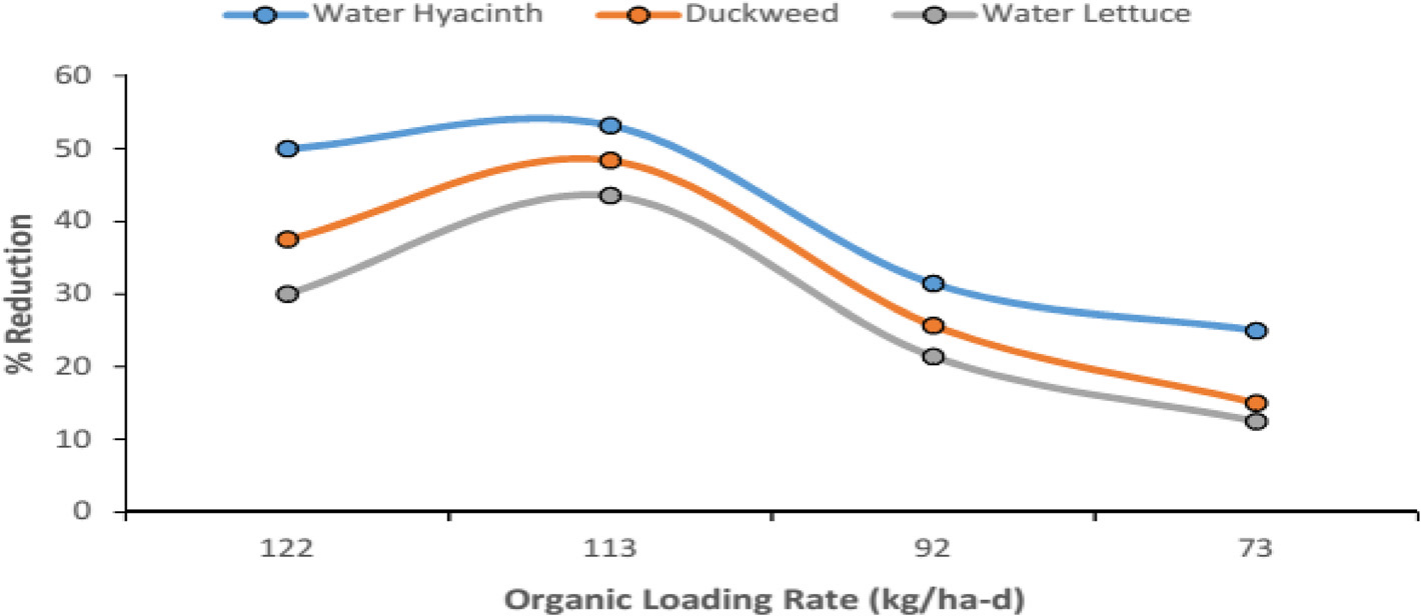
BOD removal efficiency versus OLR.

BOD removal efficiencies was also observed against Organic Loading rate (OLR). The removal efficiencies resulting in different OLR are presented in Figure 5. It shows increase in removal efficiency with increase in OLR and also the optimum OLR of 112-113 kg BOD/ha-d and by further increase in OLR results in reduction of system removal efficiency.

### COD

**Figure 6:**
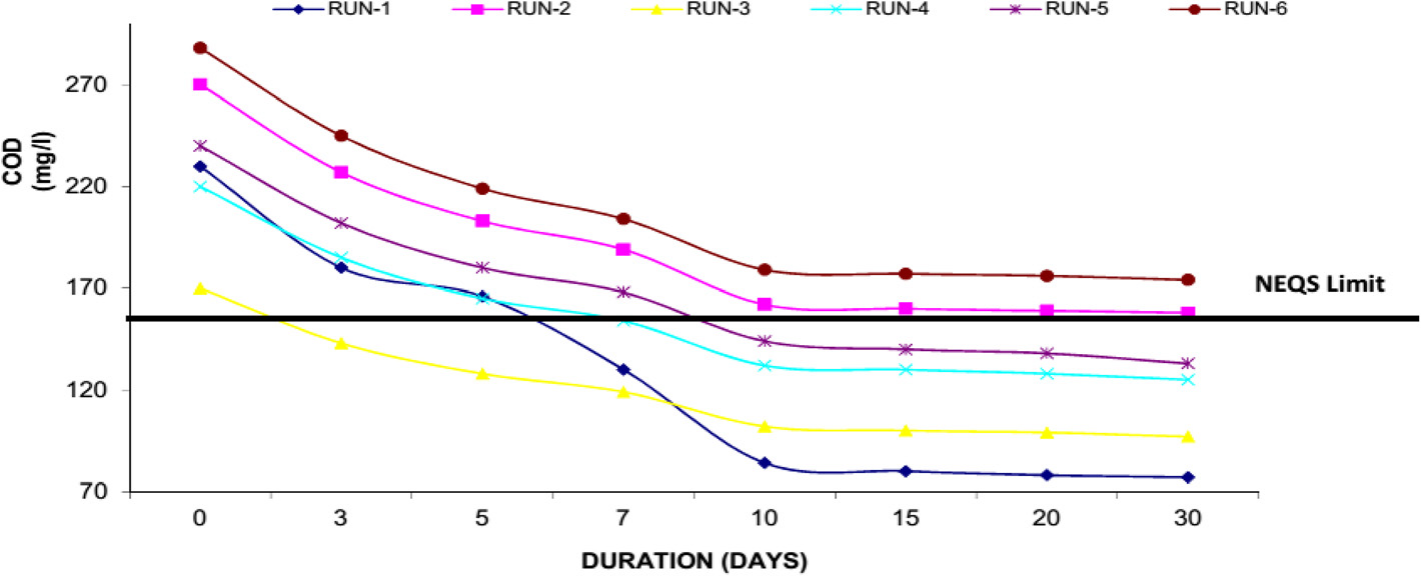
Reduction of COD with water hyacinth.

**Figure 7:**
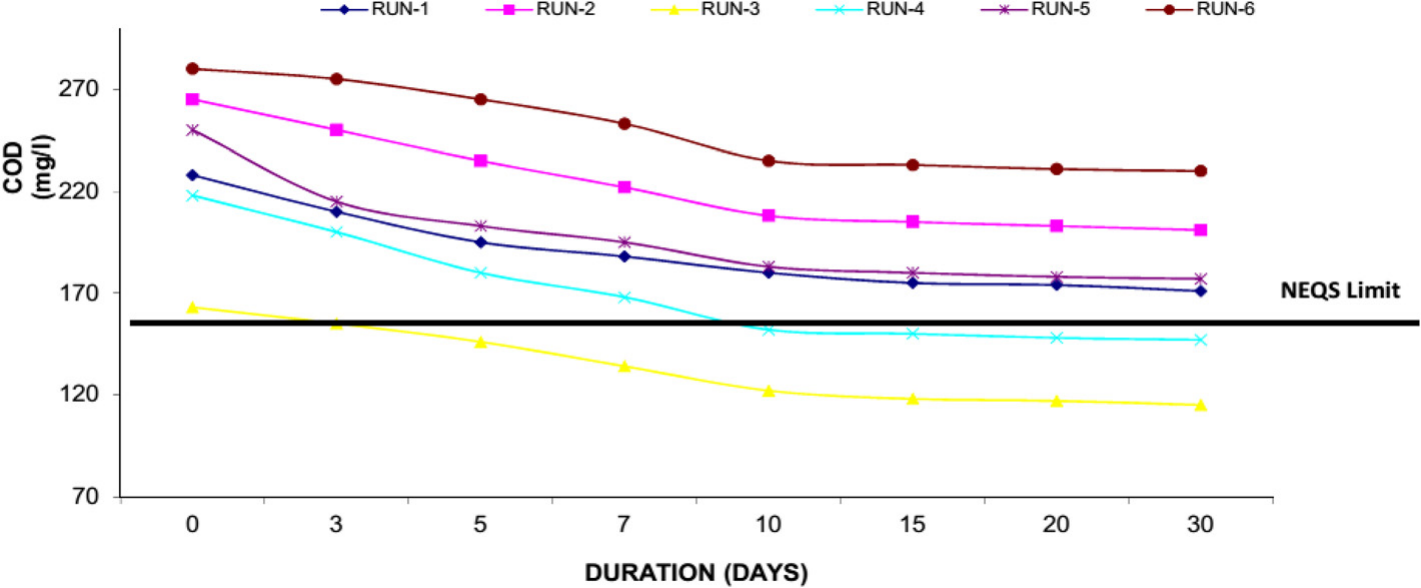
Reduction of COD with duckweed.

**Figure 8:**
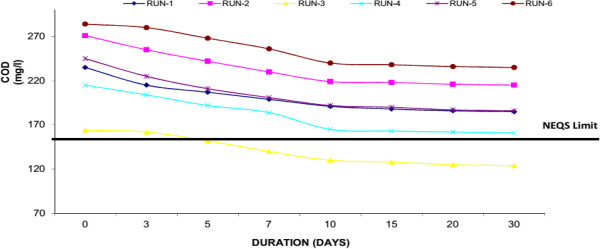
Reduction of COD with water lettuce.

COD is the amount of oxygen necessary to oxidize the Organic Compound (OC) completely to CO_2_, H_2_O and NH_3_. COD is measured via oxidation with potassium dichromate (K_2_Cr_2_O_7_) in the presence of sulfuric acid and silver and is expressed in mg/l. Thus, COD is a measure of the O_2_ equivalent of the organic matter as well as micro-organisms in the wastewater. If the COD value is much higher than the BOD value, the sample contains large amounts of organic compounds that are not easily biodegraded. Water Hyacinth was capable to decrease COD from its initial value to final value below National Environmental Quality Standards (NEQS), Pakistan i.e. <150 mg/L (50.61% average reduction). Similarly in Duckweed, it was observed that reduction of COD from initial value of 130 mg/l to final 87 mg/l (33.43% average reduction) and in Water Lettuce, the COD was reduced from initial 131 to final 94 mg/l (28.59% average reduction) respectively. Around 30-40% of decrease in the parameters occurred within the first 10 days of the experiment. Result confirmed that the growth of macrophytes and showed high performance to remove COD mainly because of well-developed root system. Similarly it was observed that a major part of the degradation of COD in the wastewater is attributed to micro-organisms which may have established a symbiotic relationship with the plants. Figures 6, 7 & 8 shows removal of COD by Water Hyacinth, Duckweed and Water Lettuce respectively.

### Ammonia nitrogen (NH_3_-N)

**Figure 9:**
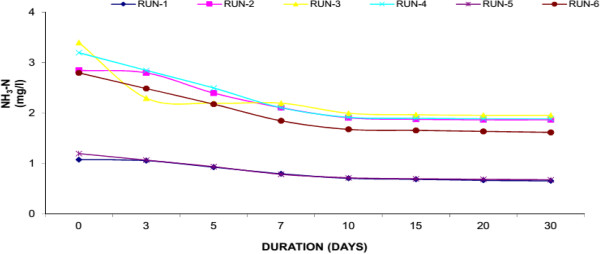
Reduction of ammonia nitrogen with water hyacinth.

**Figure 10:**
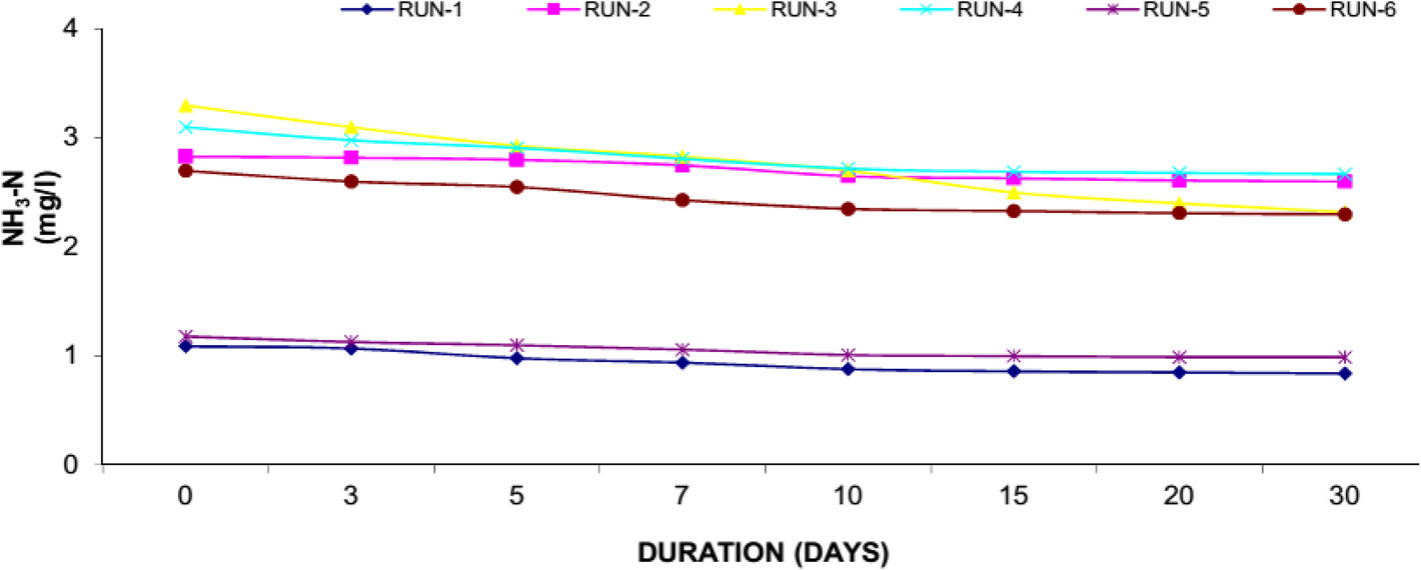
Reduction of ammonia nitrogen with duckweed.

**Figure 11:**
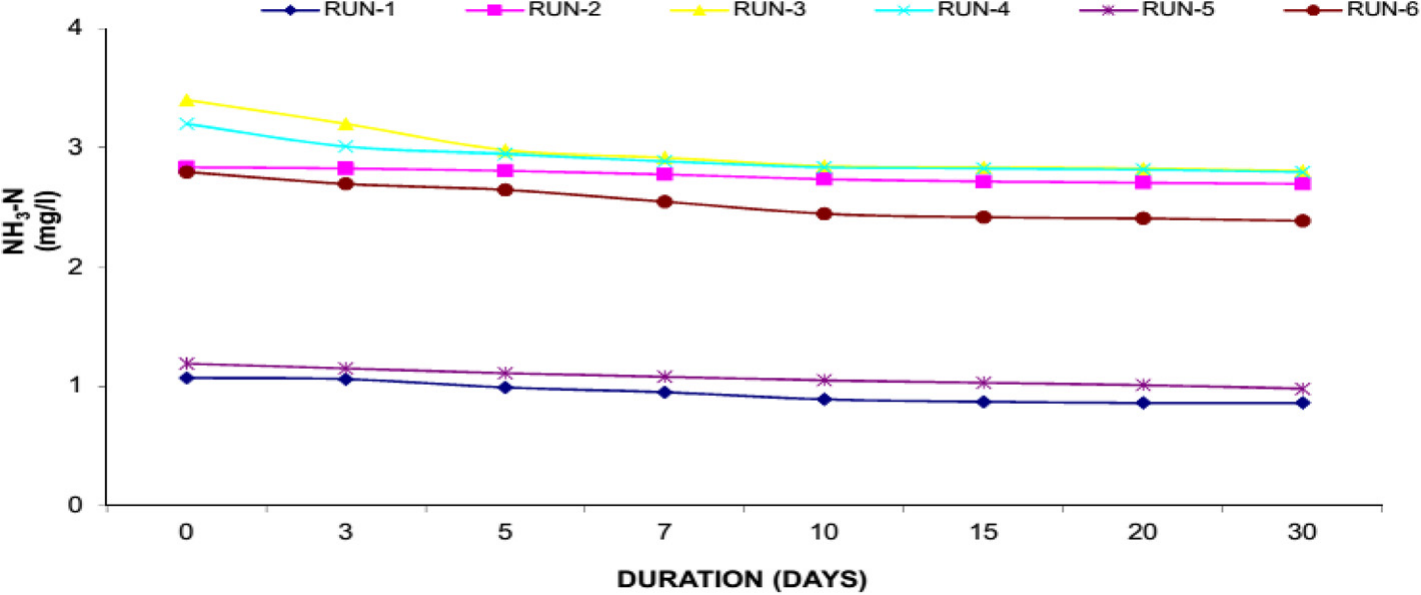
Reduction of ammonia nitrogen with water lettuce.

Urban wastewater contains high concentration of nutrients in addition to other pollutants. The major nutrients found in wastewater are N and P, which if not treated would cause number of problems to the environment especially receiving water bodies. Excess of nutrients in water body cause overproduction of phytoplankton and resulted in O_2_ depletion. N is an essential nutrient and it enters a wetland in particulate and dissolved inorganic and organic forms . Particulate forms of N are removed through a series of process including ammonification, nitrification, denitrification and ammonia volatilization. In fresh sewerage, about 25% of the “N” is in the organic form and 75% in the ammonium form. The organic nitrogen fraction is converted almost entirely to NH_3_-N, and further converted to nitrate- N (NO_3_-N) via microbial oxidation. Like COD, we have measured the N removal by different set of experiments. In Water Hyacinth system, the reduction of N ranging from 2.42 to 1.45 mg/l (40.34% average reduction) whereas the reduction in Duckweed ranges from 2.37 to 1.95 mg/l (17.59% average reduction) while in Water Lettuce reduced N from 2.42 to 2.09 mg/l (14.45% average reduction). The Water Hyacinth showed the highest level of N removal as compared to Duckweed and Water Lettuce. Result of present study showed that plants are significantly more efficient to remove N. In addition to plant uptake, N removal can occur by NH_3_ volatilization favoured by high pH, nitrification under aerobic conditions and denitrification under anaerobic conditions and formation of organic films. In the present study, N removal was occurred by volatilization because pH was higher than 6.5. It has been observed that that a greater ratio of plant biomass to model volume can enhance the contact between plant roots and wastewater resulting in a greater nutrient removal.Figures 9, 10 and 11 shows the removal of Nitrogen achieved in the study. At the end of experiment the presence of plants significantly reduced the ammonia-N from their initially levels. General decline of ammonia- nitrogen was found in all the experimental set up to 10 days thereafter the reduction is much lesser. Reduction by Water hyacinth is much more than Duckweed and Water Lettuce.

### Phosphorus (PO_4_^-3^)

**Figure 12:**
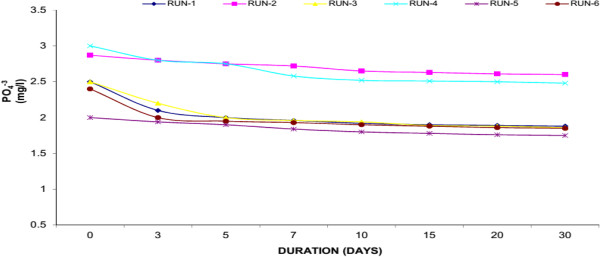
Reduction of phosphorus with water hyacinth.

**Figure 13:**
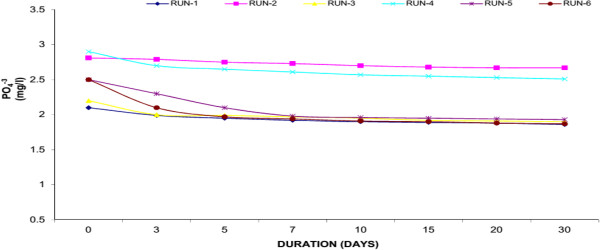
Reduction of phosphorus with duckweed.

**Figure 14:**
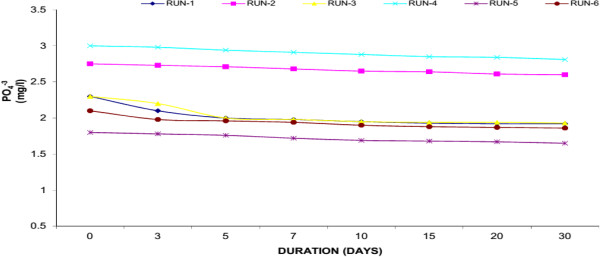
Reduction of phosphorus with water lettuce.

Phosphorus (P) is an essential nutrient for all life forms, and is the eleventh-most abundant mineral in the earth’s crust. P is needed for plant growth and is required for many metabolic reactions in plants and animals. Organic phosphorus (OP) is a part of living plants and animals, their by-products, and their remains. Generally, P is the limiting nutrient in freshwater aquatic systems. That is, if all P is used, plant growth will cease, no matter how much N is available. P typically functions as the “growth-limiting” factor because it is usually present in very low concentrations. The natural scarcity of phosphorus can be explained by its attraction to organic matter and soil particles. Any unattached or “free” P is quickly removed from the aquatic system by aquatic plants. Excessive concentrations of P can quickly cause extensive growth of aquatic plants. A normal adult excretes 1.3 - 1.5 g of P per day. Primary treatment removes only 10% of the P in the waste stream; secondary treatment removes only 30%. Tertiary treatment is required to remove additional P from the water. The amount of additional P that can be removed varies with the success of the treatment technologies used. Available technologies include biological removal and chemical precipitation.Figures 12, 13 and 14 showed the removal of P in Water Hyacinth, Duckweed and Water Lettuce based systems respectively. Water Hyacinth showed maximum removal of P (18.76% average reduction) whereas Duckweed showed 15.25% average reduction and Water Lettuce showed 10.69% average reduction within the 30 days experimental period. The highest removal was observed inWater Hyacinth. This was due to synergistic effect of aquatic plant. Plants and micro-organisms all utilize P as an essential nutrient, and contain P in their tissues though the portion of tissue P is very small compared with C and N. Reduction of T-P may be due to uptake of soluble P, Filtration of particulate matter through the roots, and settling. Although initial level of P was low but the plant like Water Hyacinth with a well-developed root system further purify the wastewater.

### Factors affecting the performance

#### Temperature

**Figure 15:**
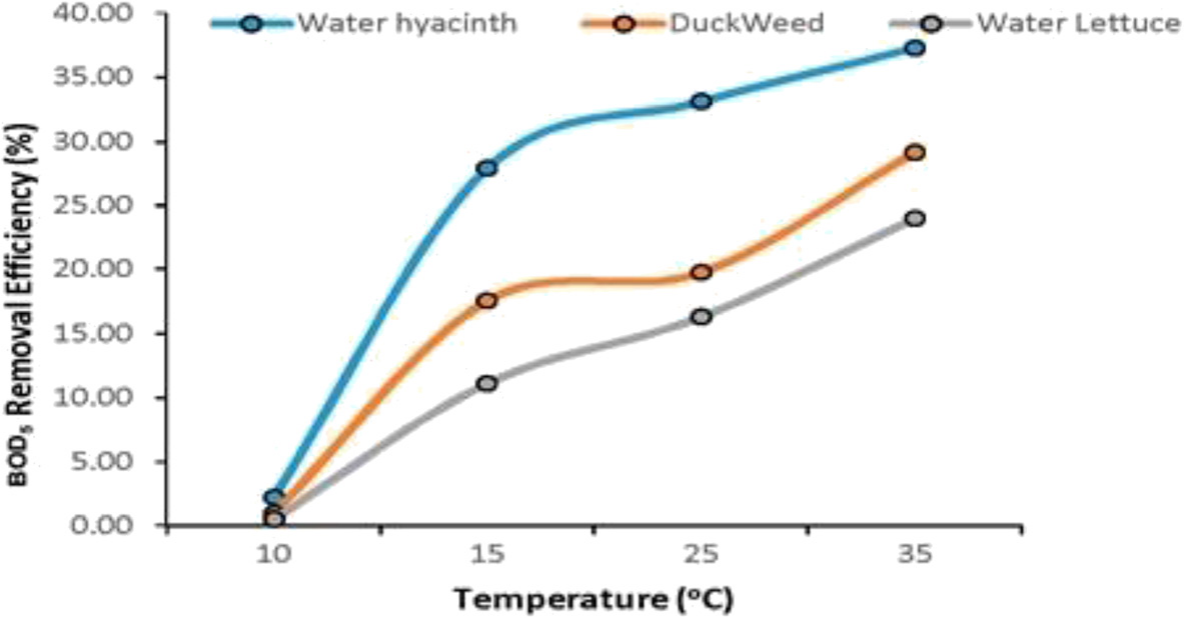
Effect of temperature variation on performance of macrophytes.

In order to check the performance of macrophytes under variation of temperature condition additional experiments were conducted with pH 7.5. Temperature has been maintained in laboratory under controlled condition at different levels to check the performance variation in treatment of macrophytes. Figure 15 shows effect of temperature variation on performance of Water Hyacinth, Duckweed and Water Lettuce regarding BOD_5_ removal. During the experiments it was observed that macrophytes are sensitive to temperature and shows no growth and pollutant (BOD_5_) removal at a temperature below 10°C. Almost all three species cease to survive at this temperature. As the growth of species is negligible at temperature below 10°C therefore, there was no uptake of nutrients (N&P) by the plants. The temperature between 15-38°C is suitable for treatment of municipal wastewater by macrophytes as high growth was observed at this temperature.

#### pH

**Figure 16:**
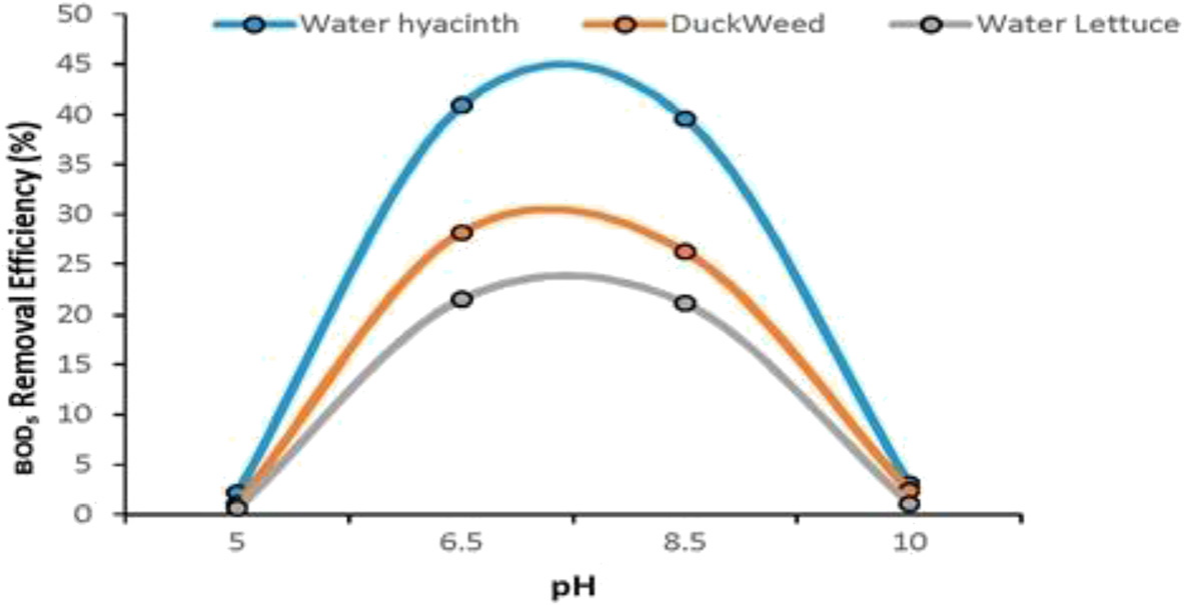
Effect of pH variation on performance of macrophytes.

In order to monitor the effect of the variation of pH on performance of macrophytes experiments in laboratory were conducted at a Temperature of 25°C and at different pH. At a pH below 5 macrophytes performance (BOD_5_ removal) in almost zero. This is mainly due to highly acidic nature of the wastewater. On the other hand when pH gradually increase performance improves upto 7.5 and by further increase in pH again start retarding macrophytes performance (BOD_5_ removal) and at a pH of 10 (at high Alkalinity) the performance of macrophytes was again decrease to zero. Therefore, a pH of 6-9 is most suitable for macrophytes performance. Figure 16 shows the effect of pH variation on the performance of Water Hyacinth, Duckweed and Water Lettuce regarding BOD_5_ removal.

### Plants growth

The plant stems filtered and reduced some particles in wastewater. When they died, they acted as net that filtered some filthy. They reduced inflow and they induce particles accumulated or precipitated in the systems. Plant heights were observed to investigate plant growth during each trial. The measured plant heights has been given below in Table 2.

**Table 2:** Plants (Macrophyte) height measurement

**Time (days)**	**Plant height (ft)**											
	**Run1**		**Run 2**		**Run 3**		**Run 4**		**Run 5**		**Run 6**	
	**WH**	**WL**	**WH**	**WL**	**WH**	**WL**	**WH**	**WL**	**WH**	**WL**	**WH**	**WL**
0	0.65	0.51	0.74	0.53	0.69	0.54	0.70	0.55	0.62	0.61	0.62	0.67
5	0.70	0.55	0.79	0.57	0.74	0.60	0.75	0.59	0.67	0.65	0.68	0.72
10	0.78	0.60	0.87	0.61	0.81	0.66	0.82	0.65	0.75	0.72	0.76	0.79
20	0.81	0.64	0.91	0.63	0.85	0.68	0.84	0.68	0.80	0.75	0.81	0.81
30	0.83	0.69	0.94	0.65	0.87	0.70	0.86	0.70	0.84	0.77	0.83	0.84

(WH = Water Hyacinth; WL = Water Lettuce).

### Plants biomass productivity

**Figure 17:**
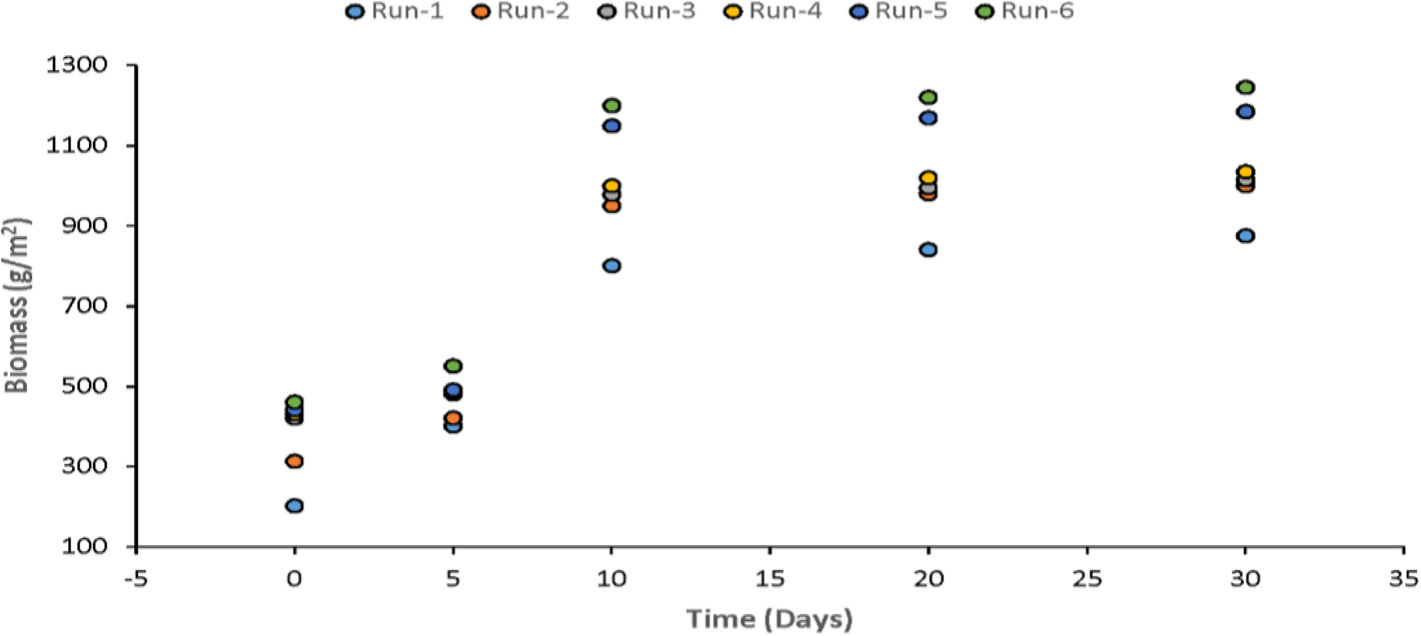
Growth of water hyacinth in study model.

**Figure 18:**
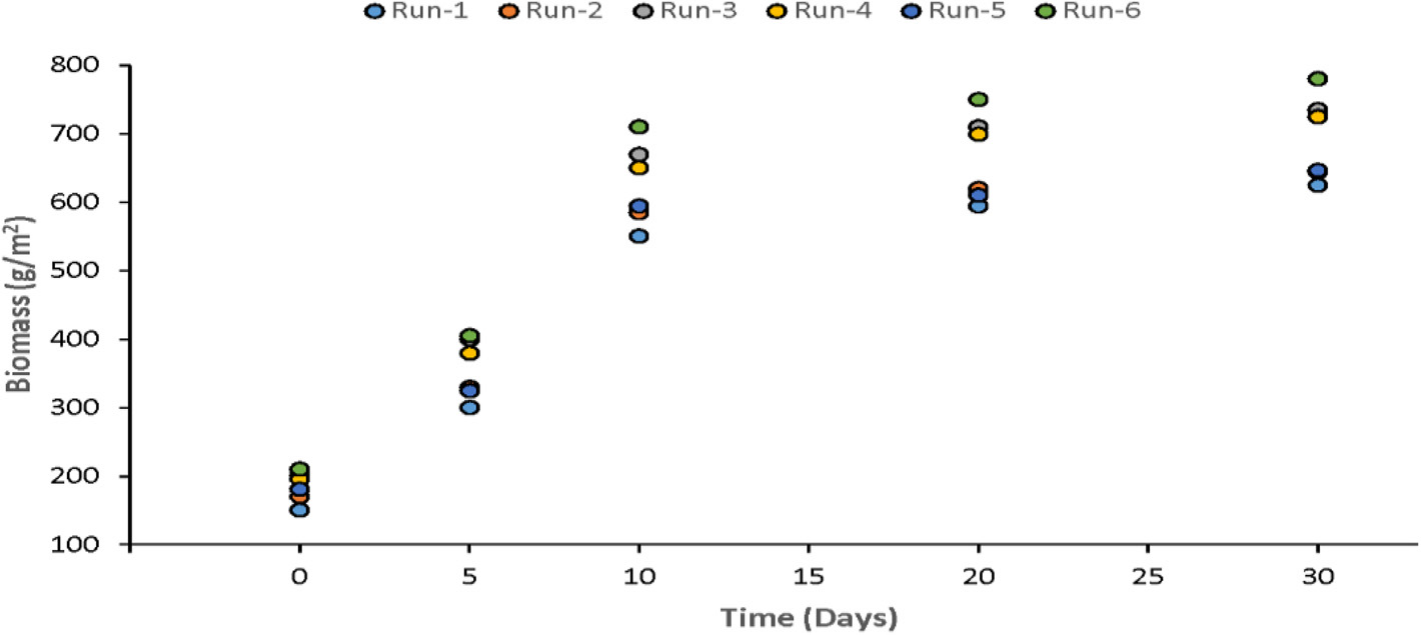
Growth of water lettuce in study model.

**Figure 19:**
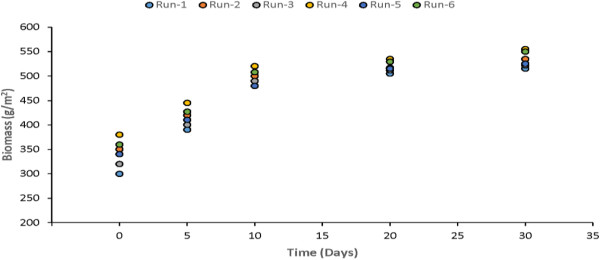
Growth of duckweed in study model.

Primary productivity and biomass are the important parameters. In general, the productivity of macrophytes is higher than that of terrestrial communities and agricultural crops because they do not suffer from shortage of water. Macrophytes have high tolerance for the fluctuations in environment conditions and show high photosynthetic efficiencies. Uptake of nutrients by macrophytes is an essential for their growth and reproduction. The high productivity of macrophytes enables substantial amounts of nutrients to be stored in plant biomass. Measurements of biomass were made after 5th, 10th, 20th and 30th day of each experimental run for Water Hyacinth, Water Lettuce and Duckweed. The plants biomass growth in the model was plotted and shown in Figures 17, 18 & 19. It is quite clear from the results that there is major rise in plants biomass in first ten however, in remaining twenty days it much less as compared to initial growth.

The results showed that the optimum period for harvesting can be found to be 8-10 days. At the optimum point, the growth rate of the plant is lowest.

## Conclusion

As far removal efficiencies are concerned Water Hyacinth is found most effective while considerable removals of pollutants were also found with Duckweed and Water Lettuce. Performance of Water Hyacinth based system was found to be 50.61% for BOD_5_, 46.38% for COD, 40.34% for Nitrogen and 18.76% for Phosphorus. For Duckweed based system the efficiencies were 33.43% for BOD_5_, 26.37% for COD, 17.59% for Nitrogen and 15.25% for Phosphorus. Similarly for Water Lettuce 33.43% for BOD_5_, 26.37% for COD, 17.59% for Nitrogen and 15.25% for Phosphorus. The mechanisms of pollutant removal in the system include both aerobic and anaerobic microbiological conversions, sorption, sedimentation, volatilization and chemical transformations. pH for the wastewater affect the performance of macrophytes and it was found that macrophytes gave optimum performance at pH 6-9. Temperature is another factor that severely affect macrophytes performance and it was found a Temperature below 10°C macrophytes were unable to perform treatment and the favorable temperature for treatment is 15-38°C. the growth of macrophytes is more in first ten days of an experimental run because in first ten days maximum treatment to wastewater is provided by macrophytes by up-taking the organic matter. Pre-treatment of wastewater before the plant acclimatization could be potentially effective. The option of macrophytes for treatment of Municipal sewage under local environmental conditions can be explored by further verifying the removal efficiency under variation of different environmental conditions. Also this is need of time that macrophyte system should be used for treatment of wastewater because their performance is comparable to conventional wastewater treatment plants and also the system has very low O&M costs.

## Competing interests

The authors declare that they have no competing interests.

## Authors’ contributions

This research is a part of the thesis by MS who prepared the literature survey and performed the experiments. AA and ARG participated in the design of the study, data analysis, and manuscript preparation. HNH was the advisor. All the authors have read and approved the final manuscript.
